# A dangerous exercise

**DOI:** 10.1186/2045-7022-5-S3-P104

**Published:** 2015-03-30

**Authors:** Thomas Medveczky

**Affiliations:** 1 Guys and St Thomas' Hospitals London, London, UK

## 

Exercise induced anaphylaxis and its subtype the Food-dependent exercise-induced anaphylaxis are uncommon and therefore under-diagnosed forms of physical allergy. Triggers include various degrees of exercise in combination with ingestion of specific food products. Treatment remains identical to that of Immunoglobulin E- mediated allergic reactions. The presentation is commonly under diagnosed and this case should raise the awareness of the attending Allergist/Physician.

A 30 year old female was seen in the Allergy Clinic upon request of her General Practitioner. She reported an episode where after dinner she went into a local park to exercise. Shortly thereafter she collapsed with rash, lip swelling and breathing difficulties. Upon admission to hospital she was found to be hypotensive and required fluid resuscitation, systemic corticosteroids and adrenaline. She made a full and eventful recovery. Based on the clinical story and the subsequent specific allergy markers her presentation was attributed to food- induced exercise-induced anaphylaxis (FD-EIA).

## Investigations in Clinic

Skin testing:

- Shellfish (mussels, clams, prawns) 4 mm

- Wheatflour 4 mm

- Milk 2 mm

- Eggs 2 mm

- positive control 5 mm

which at that time offered the possible diagnosis of either allergic reaction to omega-5-gliadin (wheat) or prawns given the skin test was positive according to international standard norm (>3mm).

Following that further serological tests were organised which revealed:

- ImmunoCAP Specific IgE blood test

- Omega-5-gliaidin allergen 0.03 kU/ml

- Shellfish 2.00 kU/ml

